# Serum Levels of Vitamin D in Diabetic Patients With and Without Retinopathy

**DOI:** 10.18502/jovr.v15i2.6734

**Published:** 2020-04-06

**Authors:** Mehrdad Afarid, Naghme Ghattavi, Mohammadkarim Johari

**Affiliations:** ^1^ Poostchi Ophthalmology Research Center, Department of Ophthalmology, School of Medicine, Shiraz University of Medical Sciences, Shiraz, Iran; ^2^ Student Research Committee, Shiraz University of Medical Sciences, Shiraz, Iran

**Keywords:** Diabetes Mellitus, Diabetic Retinopathy, 25-Hydroxy Vitamin D

## Abstract

**Purpose:**

To evaluate the levels of vitamin D in the serum of diabetic patients with and without diabetic retinopathy (DR).

**Methods:**

Thirty patients with DR and thirty diabetic patients without retinopathy were included in this cross-sectional study. Based on ophthalmic examination, patients with DR were categorized into having non-proliferative retinopathy (NPDR) and proliferative retinopathy (PDR). Patients were tested for fasting blood sugar (FBS), hemoglobin A1C (HbA1C), 25-hydroxy vitamin D (25 (OH) D), and creatinine levels in the serum, and for urine protein. Vitamin D deficiency was defined as a serum 25 (OH) D level < 20 ng/mL.

**Results:**

We found that all diabetic patients had mild vitamin D deficiency (serum 25 (OH) D level = 10–20 ng/mL). The mean serum 25 (OH) D concentration in patients with DR was lower than in those without DR (12.10 ± 14.62 ng/mL vs 15.61 ± 9.40 ng/mL, respectively, *P* = 0.012). Trace or more proteinuria was frequently present in patients with DR than in those without DR (56% in DR vs 30% in non-DR; *P* = 0.037). There were no significant differences in the FBS, HbA1C, and serum creatinine levels between patients with or without retinopathy.

**Conclusion:**

The present study demonstrated that patients with DR had lower levels of serum vitamin D compared with those without retinopathy.

##  INTRODUCTION

The prevalence of diabetes mellitus, a metabolic syndrome with several complications, has increased worldwide in the recent decades.^[1]^ Based on the latest global burden of disease estimates in 2011 (SuRFNCD-2011), the prevalence of type 2 diabetes in the Iranian population is 11%.^[2]^

Diabetic retinopathy (DR) is a major complication of diabetes mellitus that can result in retinal vascular abnormalities and severe visual impairment. DR is defined as a vascular disease resulting from hyperglycemia, and is characterized by altered structure of retinal endothelial vessels and disrupted blood–retinal barrier. The retinal vasculature is gradually damaged resulting in ischemic changes that lead to the formation of new vessels and its consequences.^[3]^

Different studies have reported abnormalities in the metabolism of calcium, phosphate, and vitamin D in diabetic patients. Bayani et al showed that vitamin D concentration was significantly lower in diabetic patients compared with that in healthy individuals.^[4]^ Moreover, animal and human studies have demonstrated a correlation between vitamin D deficiency and impaired insulin production and secretion as well as insulin sensitivity.^[5,6,7]^ Another study reported that reduced serum insulin levels could be related to the lower level of 1,25(OH) ${}_{2}$ D.^[8]^

Vitamin D has an inhibitory effect on vascular angiogenesis.^[9]^ It is believed that vitamin D deficiency, which is described as a serum 25 (OH) D concentration < 20 ng/mL, affects the pathogenesis and progression of DR.^[10]^

Vitamin D deficiency is a common public health concern. A study reports that the prevalence of vitamin D deficiency varies across different populations.^[11]^ The prevalence of vitamin D deficiency in the United States was reported to be 41.6% in 2005.^[12]^ Additionally, individuals who had no college education and had low serum levels of high-density lipoprotein cholesterol, high body mass index (BMI), hypertension or were on low calcium diet had low serum vitamin D levels.^[12]^ A study in Tehran, Iran reported the prevalence of vitamin D deficiency to be 43.3% among adolescents, with higher rates in females compared to males.^[13]^

The most likely crucial role of Vitamin D related to diabetic pathogenicity and its main complications such as DR is its involvement in several body mechanisms such as adjustment of blood glucose or body vascular consistency. Therefore, we decided to investigate the serum levels of vitamin D in our diabetic patients with and without DR in Fars Province, Iran.

##  METHODS

This cross-sectional study was conducted on patients with type 2 diabetes who were referred to the ophthalmology clinics of the Shiraz University of Medical Sciences. The study was approved by the Shiraz University of Medical Sciences Ethics Committee. Thirty patients with DR and thirty patients without retinopathy were included. Patients with a history of smoking, chronic kidney disease, active infection, liver disease, primary or secondary hyperparathyroidism, serum creatinine levels > 2 mg/dL, and those receiving vitamin D, calcium supplements, or any other medication that could change vitamin D metabolism, such as rifampin or phenytoin, were excluded from the study. Patients who had a history of bone fracture or orthopedic surgeries during the past year were also excluded.

Age, sex, and duration of diabetes were recorded for all subjects. Patients were categorized into two groups based on the duration of diabetes ( < 10 years and > 10 years).

All patients underwent complete ophthalmic examination by a vitreoretinal surgeon for diagnosing DR; this examination consisted of funduscopy with a slit-lamp and 90 D lenses and an indirect ophthalmoscopy. DR was diagnosed in the presence of one or more of the following signs: microaneurysms, cotton-wool spots, intraretinal hemorrhages, or macular edema. DR was categorized into mild to severe non-proliferative diabetic retinopathy (NPDR) based on the absence of any neovascularization and proliferative diabetic retinopathy (PDR). Mild NPDR was defined as having only microaneurysms, whereas severe NPDR was defined as having any one of the following features: severe intraretinal hemorrhages in four quadrants, venous beading in two or more quadrants and intraretinal microvascular anomalies (IRMAs) in one or more quadrants; moderate NPDR was between the above two categories. In cases of asymmetrical retinopathy, the patient was assigned to the group corresponding to the eye with the more severe retinopathy scale.

Blood samples were drawn in the morning after a 12-h fast. Fasting blood sugar (FBS) and glycated hemoglobin A1C (HbA1C) and serum calcium, phosphorus, and creatinine levels were measured. The presence of protein in the urine was checked with dipstick method.

Serum vitamin D level was assessed measuring the level of serum 25-OH vitamin D. The 25 (OH) D levels were measured from serum samples using solid phase enzyme-linked immunosorbent assay (DRG 25 (OH) D total ELISA Kit; DRG Instruments GMBH, Germany). The serum concentration > 30 ng/mL was considered as sufficient. Cut-offs for serum vitamin D concentration status are described in Table 1.

**Table 1:** Serum 25(OH) D concentration^[14]^


**25(OH) D concentration**	**25(OH) D status**
≤ 10 ng/mL	Severe deficiency
10–20 ng/mL	Mild deficiency
20–30 ng/mL	Insufficient
30 ≤ ng/mL	Adequate
100 ≤ ng/mL	Potential toxicity

**Table 2:** Demographic and clinical characteristics of patients


	**Patients with no DR**	**Patients with DR**	***P*** **-value**
Gender (female/male), n	16/14	15/15	0.73
Age (years), mean ± SD	58.30 ± 7.23	58.57 ± 5.78	0.75
FBS (mg/dL), mean ± SD	146.43 ± 58.91	164.37 ± 88.98	0.54
HbA1c (%), mean ± SD	7.18 ± 1.58	7.90 ± 2.25	0.45
Hb (g/dL), mean ± SD	14.20 ± 1.22	13.48 ± 2.04	0.78
BUN (mg/dL), mean ± SD	13.66 ± 3.98	17.03 ± 7.40	0.41
Cr (mg/dL), mean ± SD	1.02 ± 0.25	1.21 ± 0.72	0.33
Calcium (mg/dL), mean ± SD	9.26 ± 0.45	8.92 ± 0.48	0.511
Phosphorus (mg/dL), mean ± SD	3.75 ± 0.42	3.66 ± 0.50	0.416
25-OH D (ng/mL) mean ± SD	15.61 ± 9.40	12.10 ± 14.62	0.012
BUN, blood urea nitrogen; Cr, creatinine; DR, diabetic retinopathy; FBS, fasting blood sugar; HbA1c, hemoglobin A1c; Hb, hemoglobin; SD, standard deviation.		

### Statistical Analysis

SPSS 17.0 software for Windows (SPSS Inc., Chicago, IL, USA) was used for data analysis. The variables with a normal distribution are shown as mean ± standard deviation, and nominal variables are expressed as number and percentage. The normality assumption in continuous variables was evaluated using the Kolmogorov–Smirnov test. Distributions higher than *P* > 0.05 were accepted as normally distributed variables. The differences between normally distributed and continuous independent variables were assessed by the independent samples *t*-test. Those without a normal distribution were compared using the Mann–Whitney U test between the groups. Categorical variables were compared using the Chi-squared test. For all comparisons, *P* < 0.05 was considered significant.

##  RESULTS

The study included 15 female subjects with mean age ( ± standard deviation) of 60.13 ± 6.02 years and 15 males with mean age of 57.60 ± 5.43 years in the group with DR and 16 females with mean age of 59.05 ± 6.25 years and 14 males with mean age of 57.30 ± 8.50 years in the group without DR. There was no statistically significant difference between the groups regarding their age (*P* = 0.73) and sex (*P* = 0.75).

The demographic and clinical characteristics of the patients in the study are shown in Table 2.

Among patients with DR, 21 patients had NPDR (13 mild to moderate and 8 severe) and 9 patients had PDR. All patients had mild vitamin D deficiency. The mean serum vitamin D concentration in patients with DR (12.10 ± 14.62 ng/mL; NPDR and PDR) was lower than in those without DR (15.61 ± 9.40 ng/mL) (*P* = 0.012). Regression analysis with independent variables (FBS, duration of diabetes and insulin use) showed similar results (*P* = 0.031).

The mean 25 (OH) D concentration between the DR subgroups (severe NPDR and PDR vs mild to moderate NPDR) was 10.51 ± 10.45 ng/mL and 13.45 ± 8.4 ng/mL, respectively (*P* = 0.681).

Serum 25 (OH) D concentration in all patients was compared among the age groups. The mean ± SD in patients with age ranging from 41 to 50 years, 51 to 60 years, and ≥ 60 years were 10.11 ± 9.50, 14.15 ± 16.06, 14.99 ± 6.94, respectively (*P* = 0.128).

Regarding the treatment, 46% of patients without DR and 86.6% of patients with DR were taking insulin. Univariate analysis between the two groups showed that the use of insulin in patients with DR was significantly higher than in those without DR (*P* = 0.014).

The duration of diabetes was different between the groups. Nine (30%) patients with no diabetic retinopathy (NDR) had diabetes for more than 10 years and 16 (53%) patients with DR had diabetes for more than 10 years. However, the difference was not statistically significant (*P* = 0.081).

Binomial variable multiple logistic regression analysis with independent variables revealed that FBS, HbA1C, BUN, serum creatinine, and serum vitamin D levels did not show the duration of diabetes as an independent risk factor for DR (*P* = 0.067). However, this value was significant in terms of the type of treatment (insulin use or not) (*P* = 0.005).

Patients with DR had a higher positive dipstick test rate for proteinuria than those without DR (56% of the patients with DR had traces of or more proteinuria vs 30% in no DR; *P* = 0.037).

FBS, serum BUN, and creatinine levels were not different between the patients with or without retinopathy. Although vitamin D may influence the levels of serum calcium, our data showed no significant difference in serum calcium or phosphorus between the two groups (*P* = 0.511 and *P* = 0.416, respectively).

##  DISCUSSION

In the current study, we evaluated the serum levels of vitamin D in diabetic patients with and without DR. Furthermore, we investigated other possible factors that could influence the progression of DR. Similar to previous studies, all included patients had 25 (OH) D deficiency.^[15,16,17]^ The current study showed that the mean serum 25 (OH) D concentration in patients with DR was lower than in those without DR, especially those with severe NPDR and PDR. These results were comparable to other studies. For example, Payne et al found that diabetic patients, especially those with PDR, had lower 25 (OH) D levels than those without diabetes.^[19]^ Similarly, Luo et al in their meta-analysis that included eight studies involving 13,435 participants showed vitamin D deficiency (serum 25 (OH) D levels < 20 ng/mL) increased the risk of DR (OR = 2.03, 95% CI: 1.07–3.86)^[20]^ On the contrary, some studies found no differences in serum vitamin D levels between diabetic patients with and without retinopathy.^[16,18]^

Evidence shows that vitamin D may affect the pathogenesis of DR via its effects on angiogenesis by changing the presence of hypoxia-inducible products, such as vascular endothelial growth factor (VEGF).^[9]^ Ben-Shoshan et al found that 1, 25 (OH) ${}_{2}$ D ${}_{3}$ decreases the protein expression of both regulated hypoxia-inducible factor (HIF)-1α subunit and the VEGF in human cancer cells.^[21]^ Vitamin D reduces inflammatory products by decreasing the lymphocyte proliferation and cytokine production.^[22]^ Moreover, vitamin D deficiency influences the activity of tissue matrix metalloproteinase (MMPs) and C-reactive protein (CRP) that are involved in microangiopathies.^[23]^

We demonstrated that DR occurred more frequently in patients who were on insulin therapy than in those who used oral hypoglycemic agents to control their blood sugar (*P* = 0.005). This could be attributed to low vitamin D concentration in these patients that could disturb the regulation of internal insulin secretion and increase the need for external insulin to control the blood sugar.^[7,8]^ In the present study, there were no statistically significant differences between groups regarding their HbA ${}_{1}$ c, FBS, and the duration of diabetes. Furthermore, patients with DR had higher proteinuria than those without, which could demonstrate a correlation between DR and nephropathy.

Multiple studies reported that the presence of DR in type 2 diabetic patients was associated with longer duration of diabetes and higher levels of HbA ${}_{1}$ c.^[24,25,26]^ However, in the present study, such correlation was not found; this disagreement could be explained by the small sample size of the current study that was not sufficient to detect these associations.

The cross-sectional design and the small sample size are the limitations of our study. Furthermore, outdoor activity time, patients' daily diet, and the mean blood pressure of patients, which are known to affect vitamin D levels,^[27]^ were not considered in the study.

Although there are several published studies on this topic with different results, to the best of our knowledge, no study on this subject is available from Fars Province, Iran. To improve our understanding of vitamin D deficiency and DR, a larger population-based study is required.

In conclusion, the current study showed that patients with type 2 diabetes and DR had lower serum vitamin D levels than those without DR.

##  Financial Support and Sponsorship

Shiraz University of Medical Sciences.

##  Conflicts of Interest

There are no conflicts of interest.

##  Acknowledgement

The authors are grateful to Dr. Kazem Kamran, retinal specialist, and Dr. Roustsa Statistician, and all staff members of the Poostchi Ophthalmology Research Center involved in the study that contributed to the recruitment of participants and supported our activities.
